# Ecological Dynamics as an Accurate and Parsimonious Contributor to Applied Practice: A Critical Appraisal

**DOI:** 10.1007/s40279-024-02161-7

**Published:** 2024-12-21

**Authors:** Dave Collins, Howie J. Carson, Pär Rylander, Ray Bobrownicki

**Affiliations:** 1 Human Performance Science Research Group, Institute for Sport, Physical Education and Health Sciences, The University of Edinburgh, Edinburgh, UK; 2 Grey Matters Performance Ltd, Stratford-Upon-Avon, UK; 3 Department of Food and Nutrition and Sport Science, University of Gothenburg, Gothenburg, Sweden; 4 School of Health and Human Performance, Dublin City University, Dublin, Ireland

## Abstract

With sport coaches adopting and working toward increasingly evidence-grounded approaches to practice, skill acquisition has appropriately become a critical area for consideration. As part of this growing interest in skill acquisition, the ecological dynamics approach has garnered attention amongst scholars and practitioners with myriad media (e.g. peer-reviewed articles, books, podcasts and social-media outputs) extolling its benefits. In doing this, however, the available guidance, advice and scholarship have typically positioned ecological dynamics as a direct competitor to existing or traditional cognitive approaches, advising against practical integration of approaches due to theoretical incompatibility. As a standalone approach, we are concerned that there are mechanistic and epistemological issues and inconsistencies that prevent experimental comparisons and limit its applicability, novelty and capability to comprehensively address real-world athlete and coach needs. Based on this, in this *Current Opinion* paper, we lay out these concerns and critically examine the clarity, coherence and consistency of the approach and its associated literature. In concluding, we also suggest that a more evidence-based and mechanistically driven approach that draws upon a wider set of theoretical perspectives can offer greater benefit to athletes, coaches and practitioners in real-world sport.

## Key Points

**Table:** 

There are concerns with clarity, coherence and consistency within the ecological dynamics approach and associated literature that may limit empirical investigation and translation.
Although elements of ecological dynamics may apply in some cases, current theory does not comprehensively account for key concepts that underpin established practice in sport.
Ecological dynamics alone appears insufficient to guide practice, so approaches that draw upon a wider range of theoretical perspectives may offer greater benefit to coaches and athletes.

## Introduction

Within sport-related research, there is growing awareness of the need for debate and scholarly analysis if theory is to positively translate to practice (e.g. [1]). Accordingly, recent studies have critically examined contemporary accounts of motor learning and control (e.g. [2–4]). As a discipline, this important discourse has been further stimulated by pressure to produce impactful research that is both process and outcome driven (see [5]) leading, in turn, to increasing collaboration between researchers and practitioners (e.g. in the design of studies or conceptualisation of practice frameworks; see [6, 7]). Positively, this has afforded advances within the field by offering alternative and critical perspectives toward basic/fundamental ideas (e.g. [8, 9]). Despite these advancements, however, motor learning and control theory remains at a challenging stage of transition concerning the veracity of applied implications, especially those with an empirical underpinning. Indeed, Ranganathan and Driska [10] recently highlighted that conclusions from motor control theory are often problematic owing to insufficient empirical evidence (from both laboratory-based and applied research) and limited scrutiny of associated theoretical mechanisms. As pointed out many years ago by Christina [5], evidence bases should be reconcilable against fundamental principles, applied theories and effective applied solutions. Accordingly, there is need to guide and build theories of skill acquisition that account for field-based data and real-world observations [10] to reconceptualise the nature of translational research that is both theoretically grounded *and* impactful.

One such theory of interest that has generated considerable attention in recent years—in academic literature, social media and real-world sporting circles—is ecological dynamics. Ecological dynamics represents an ‘alternative framework’ to cognitive information processing theories [11, p. 30] that combines theoretical developments from assorted domains outside of sport (e.g. motor learning, psychology and neurobiological systems) to explain the acquisition and performance of movement skills in sport. A key basis for ecological dynamics is the ‘degrees of freedom problem’, identified by Russian neurophysiologist Nikolai Bernstein [12], whereby humans are tasked with coordinating innumerable subcomponents of the movement system (e.g. from muscles and joints down to cells) to perform complex tasks, which are posited to be too many to consciously control. To manage the seemingly overwhelming complexity of available components, which outnumber the minimum quantity of components necessary to perform a task (known as redundancy), Bernstein proposed that learners initially simplify the system by freezing available degrees of freedom (e.g. by coupling muscle–joint segments) before gradually releasing them with practice amidst smoother, more economical movement and increasing variability (e.g. within and between joints), for which there has been some empirical support ([13] but see also [14]).

However, from the ecological dynamics perspective, the coordination of human movement involves more than controlling degrees of freedom alone. Instead, informed by developments from the dynamical systems approach which explains patterns encountered throughout nature [15], the ongoing interaction between organismic, task and environmental constraints determines ‘the optimal pattern of coordination and control for any activity’ for any given organism [16, p. 348]. In sport, the organismic constraints will relate to performer characteristics, including trait-like structural constraints (e.g. height and limb length) and state-like, functional constraints such as psychological characteristics (e.g. emotions and motivations). Task constraints in sport concern contextual performance-related factors such as task goals, rules (e.g. must jump off one leg in high jump), equipment (e.g. the size, weight, or tension of striking implements such as tennis racquets) or facility properties (e.g. pitch size, turf type, or boundary markings) [11]. Environmental constraints comprise physical factors from nature such as gravity, altitude, temperature, or weather conditions (e.g. rain or snow). Typically included within this environmental category are also social constraints, such as cultural behavioural norms and audience presence. With these myriad constraints, the interaction between all of these components is thought to be ever changing, unpredictable and non-linear, and because movement emerges based on this interplay, every performance varies and is, consequently, unique [17].

It is against this backdrop of inherent variability—which was inspired from work outside sport—that proponents of ecological dynamics have typically advised against several heretofore common coaching approaches, including standardised drills and training within sport (e.g. [18]), and have discounted concepts such as internal representations and knowledge as critical for movement control [19]. Instead, a key concept for coordinating these interactions between individual, task and environmental constraints relates to the coupling of perception and action, which has been adopted from ecological psychology, particularly the work of Gibson [20]. In this regard, information from the environment (e.g. from visual, haptic, or auditory sources), which is directly perceived (i.e. not interpreted or reliant on centralised processing), facilitates ‘circular relations’ between the organism and the environment to dynamically constrain behaviour [21] with learning characterised by ‘attunement’ to the most relevant perceptual information [22]. This complementary relationship can also reveal opportunities for action within the environment, known as affordances, another concept borrowed from Gibsonian [20] psychology. According to Gibson, and demonstrating the non-sport origins, ‘the affordances of the environment are what it *offers* to the animal, what it *provides* or *furnishes*’ (p. 119). For instance, for early humans, ‘an approaching rabbit afforded the opportunity to eat whereas an approaching tiger afforded being eaten’ (p. 94).

For proponents of ecological dynamics, with the importance of constraints, direct perception–action coupling, and affordances, the design and representativeness of the training environment consequently become critical considerations for coaches and practitioners [22], while developing understanding or practising skills in isolation expressly do not [23, 24]. Because of ecological dynamics’ proposed implications for practice, there has been a corresponding proliferation of advice on possible application in sport in recent years (e.g. [25, 26]) and, curiously, also applications to a growing range of new, unexpected domains (e.g. esports [27], mathematics education [28], injury risk [29] and police training [30]).

Although there is acknowledgement that ecological dynamics could, in part, account for elements of sports performance (e.g. [2]), there are concerns that its application is being overextended beyond what its evidence base can reasonably explain (see [2, 25]). Moreover, ecological dynamics theory neither comprehensively accounts for common, established and/or empirically supported aspects of training in sport (e.g. mental imagery, game plans and performance reviews), nor sufficiently explains key constructs such as cognition and intention. Indeed, with ecological dynamics’ reliance on the direct perception of information (and, thus, not on internal representations and other cognitive mechanisms), central issues in debates or comparisons with information-processing approaches are the unclear or limited contributions of cognition, intention and myriad psychological factors (e.g. understanding, confidence, motivation, etc.), which typically represent important and common considerations for coaching practice. These issues become potentially more pronounced when trying to apply ecological dynamics, an amalgamated theory of motor learning that embodies or minimises the contribution of the brain and cognition, to practice in other domains such as mathematics, learning and pedagogy [31], which would ostensibly value cognition and understanding as central tenets, and would ordinarily emphasise neither perception nor action. As Ranganathan and Driska [10] pointed out, as the priorities of scholars and practitioners are not necessarily aligned, and data collection is often far removed from real-world settings, it is ‘non-trivial to translate research’ to real-world environments (p. 3) and more cautious recommendations appear warranted.

These dissonances between theory and practice may explain findings from Richardson et al. [32] that suggest practitioners in sport neither sufficiently understand nor are convinced by ecological dynamics, its underpinning mechanisms and its capability to meet expectations for effective learning and teaching. Although Richardson et al. [32] consequently challenged practitioners to understand before acting, we also put forward that it is equally important for scholars to clearly establish and understand the concepts, implications and concomitants of ecological dynamics before promoting its application. On this point, we are concerned that there are issues with clarity, coherence and consistency in the ecological dynamics literature that limit translation and require close evaluation and attention. Based on this, the purpose of this *Current Opinion* article is to extend discourse within this topic by outlining these concerns with the ecological dynamics literature to date. Specifically, we (1) identify crucial inconsistencies within ecological dynamics literature that may create confusion amongst practitioners, (2) contrast ecological dynamics-based practices with those known to be effective within learning in general, (3) examine the nature of the evidence within ecological dynamics literature, (4) challenge the apparent novelty of ecological dynamics-based practices against what good coaching has advocated for many decades, and (5) question the applicability of ecological dynamics beyond the process of skill acquisition. In laying out these concerns and critically exploring ecological dynamics, we conclude by proposing potential next steps for scholarship in this area.

## First Concern: A Lack of Conceptual Clarity

Conceptual clarity is a key component for enabling connections across studies and generating future research (see [33] for discussion of these points in sport coaching). In this regard, for theory to be tested, a fundamental component of the scientific process is the establishment of clear concepts and constructs, alongside implications or points of difference. Between and even within papers, however, there appear to be inconsistencies in ecological dynamics scholarship that limit conceptual clarity and the consequent capability for testable hypotheses. In part, some of these conceptual inconsistencies could relate to the disparate origins that have informed ecological dynamics’ development, as myriad researchers have only later tried to fit these concepts to sport. To illustrate the inconsistencies, we examine three important components (i.e. mental representations, cognition and knowledge) using papers authored by proponents of the ecological dynamics approach.

### Inconsistencies ‘Between’ Authors

Examining between articles, quotations in the left-hand column of Table 1 represent clear ‘absolute’ positions expressed in the literature, whereas those in the right-hand column are more ambivalent. Regarding mental representations, for example, a primary, traditional and consistently contentious target for ecological dynamics, both Handford et al. [34] and Seifert et al. [35] clearly dismissed the necessity of centralised structures for interpreting information or control movements. In contrast, Araújo et al. [19] more equivocally stated that behaviours can be controlled in a way not *necessarily* requiring mental representations, despite also stating in ecological dynamics ‘there is *no* [emphasis added] internal knowledge structure or central pattern generator inside the organism responsible for controlling action’ (p. 10). Equally confusing, Correia et al. [36] recently stated there was ‘little focus’ (p. 121) on strengthening mental representations, which seems to not dismiss representations entirely.

**Table 1:** Between-author inconsistencies concerning representation, cognition and knowledge within the ecological dynamics literature in the skill acquisition domain

Construct	Exemplar “absolutist” perspective no. 1	Versus	Exemplar “vague” perspective no. 2
Representation	Handford et al. [34]—‘Direct realists do not subscribe to the view that biological organisms need highly developed, detailed internal representations to ascribe meaning to the information supplied by the sensory systems.’		Correia et al. [36]—‘periods following the micro-structure of practice are not as reflexive in mental rehearsal as in traditional pedagogical approaches. There is little focus on strengthening mental representations or on reinforcing explicit knowledge of an activity.’
	Seifert et al. [35]—‘A movement is not an entity stored by an individual in the central nervous system, but rather a dynamically varying relationship resulting from the constraints imposed by the environment and the resources of a performer.... an individual’s expertise can be explained without postulating internal controlling structures.’		Araújo et al. [19]—‘Behaviours can be sustained by simultaneous and successive affordances, and not necessarily by a hierarchical plan or representation capturing a sequence of performance operations’
Cognition	Silva et al. [39]—‘we challenge the concepts of shared knowledge and team cognition and propose that team coordination is, rather, predicated on shared affordances... The concept of affordances presupposes that the environment is perceived directly in terms of what an organism can do with and in the environment (i.e., it is not dependent on a perceiver’s expectations, nor mental representations linked to specific performance solutions, stored in memory).... players can detect information from patterned energy arrays in the environment in terms of their own characteristics (e.g., individual height, in basketball) [43] or in terms of their action capabilities (e.g., perceiving a defender’s most advanced foot invites the attacker to drive an attack to that side) [44]. This information constrains behaviour by providing affordances or behavioural possibilities for decision making.’		Passos et al. [41]—‘Although an individual may use previous experiences to consider a ‘ball park’ performance outcome solution, in team games an opponent’s specific movements form a major task constraint that shapes emergent decision-making behaviour in each individual performer.’
	Chow et al. [40]—‘These advances in embodied cognition emphasize the learner–environment relationship.... influenced by concepts in ecological psychology and nonlinear dynamics such as information–action coupling, self-organization, constraints, emergence, variability and stability of behavior in neurobiological systems... it has been shown how complex neurobiological systems continuously adapt and change their organizational states through processes of spontaneous self-organization... In nonlinear neurobiological systems, constraint configurations do not prescribe each learner’s behavior but simply guide it through interaction with his/her perceptual-motor systems.’		Correia et al. [36]—‘at very early stages of learning a functional action pattern may not exist and instructions with an internal attentional focus may direct learners to the specific part of an affordance landscape which needs to be searched in practice to help them explore relevant functional performance solutions (Peh, Chow, and Davids 2011). For instance, a tennis learner who performs the backhand volley much at the expense of flexion extension of the arm, could be asked to seek to keep the arm in extension in front of the trunk and perform the backswing essentially through rotation of the trunk.’
Knowledge	Davids [42]—‘skilled performance gradually derives from the increasingly improved (functional) fit of an individual and an environment, rather than from an increased complexity of acquired knowledge and associated computational and memorial processes.’		Passos et al. [41]—‘the first step to face the problem is to detect action possibilities; this could be done with off-field manipulations. For example, video analysis is a powerful tool to recognize patterns of play and detect action possibilities. Another example is the use of notational analysis, which provides statistics of opponents’ patterns of play that allow identification of strengths and weakness. Finally, there is the use of cognitive strategies such as imagery or self-talking which are useful techniques to detect and anticipate action possibilities.’
	Renshaw et al. [46]—‘This emphasis on the quality of the individual–environment relationship is exactly why the CLA could never be included under the scope of any framework of constructivism.’		Roberts et al. [44]—‘the learners in the practice environment will endeavour to make sense of the chaos they are presented with by forming performance solutions via goal-directed behaviour.... well-structured environment design must offer learners the opportunity to move beyond “what” they must do, and towards an understanding that allows them to construct for themselves the “how, why, where and when” of movement.’

Addressing a related, suggestively mediating construct in forming and activating mental representations and expressions of behaviour (e.g. [37, 38]), ‘cognition’ is also presented inconsistently in terms of relevance and role. Left-hand column quotations in Table 1 dismiss cognition within any mechanistic explanation, instead utilising alternative conceptual processes. For instance, Silva et al. [39] used terminology such as ‘affordances’ and environmental energy patterns to directly inform decision-making and behaviour. Chow et al. [40] referred to information–action coupling, self-organisation, constraints, emergence, variability and stability of behaviour as core features of embodied cognition without cognition as a central process. Contrastingly, however, Passos et al. [41] acknowledged the need to ‘consider’ different performance solutions from ‘previous experiences’ within team sports, which appears to reflect or require cognition and memory. Further adding to this inconsistency, Correia et al. [36] directly advocated the conscious control of movement kinematics during skill acquisition. So, as with mental representations, it is difficult to clearly understand the ecological dynamics position on how cognition is operationalised and/or mechanistically underpins performance and its development.

Finally, concerning the development of detailed knowledge as underpinning skill acquisition, Davids [42] explicitly contested this notion, which is further reflected in Renshaw et al.’s [43] subsequent rejection of ecological dynamics within constructivist frameworks. Again, however, this very constructivist approach is surely what Passos et al. [41] and Roberts et al. [44] described when referring to off-field video analysis to recognise patterns of play in team sports and the development of a detailed understanding of what, when, how, why and where to move in physical education (PE) contexts.

### Inconsistencies ‘Within’ Authors

This inconsistency is not limited to *between* articles; it exists *within* them too. Using the same three concepts, Table 2 presents these differences using several exemplars. Regarding mental representations, Handford et al. [34], for instance, initially dismissed these before reporting the ecological dynamics approach as ‘less focused’ (p. 625) on such control structures compared with traditional information-processing approaches. On this basis, it is uncertain whether mental representations do exist but are merely less important. More recently, Button et al. [45] first endorsed the notion of memorised movements but later dismissed mental representations. It is, therefore, difficult to understand how such memory is formed or what it constitutes.

**Table 2:** Within-article inconsistencies concerning representation, cognition and knowledge within the ecological dynamics literature in the skill acquisition domain

Construct	Statement no. 1	Versus	Statement no. 2
Representation	Handford et al. [34]—‘Direct realists do not subscribe to the view that biological organisms need highly developed, detailed internal representations to ascribe meaning to the information supplied by the sensory systems’		Handford et al. [34]—‘The ecological approach is less focused on the internalized knowledge structures or executive regulators which form an integral part of traditional information-processing theories’
	Renshaw et al. [43]—‘This emphasis on the quality of the individual–environment relationship is exactly why the CLA could never be included under the scope of any framework of constructivism.’		Renshaw et al. [43]—‘In such dynamic neurobiological systems, there is no particular component (e.g. a representation in the mind) leading/controlling the other components (the physical movement, the sport skill). The key point is that the continuous and ongoing interactions of a multitude of constraints facilitate the emergence of functional behaviours (e.g., thoughts, ideas, actions, perceptions, intentions) in each individual’
	Button et al. [45]—‘[f]or learning to occur, the performer must use the task to be learned as a specific form of information to remold the landscape. In other words, “once learning is achieved, the memorized pattern constitutes an attractor, a stable state of the (now modified) pattern dynamics” (Kelso, 1995, p. 163).’		Button et al. [45]—‘Mental representations are not needed to explain behavior in an ecological dynamics approach’
Cognition	Chow et al. [40]—‘The importance of the perceptual-motor system and how it uses information from the performance context is clearly exemplified by Jacobs and Michaels (2007) in their discussion on “Direct Learning”. The lack of dependence on inference and cognitive processing as mechanisms for the acquisition of movement skills was highlighted. Instead, an emphasis on how information from the environment, in the form of ambient energy arrays, is considered critical in channeling learners to learn movement skills’		Chow et al. [40]—‘Specifically, the learner’s intentions and attention to these informational variables change when learning occurs (see Jacobs and Michaels (2007) for further discussion). This idea of “Direct Learning”, where perceptual information is directly mapped to action, is relevant to understanding how goal-directed behaviors emerge under the confluence of various constraints in the performance context’
	Renshaw and Chow [46]—‘Despite misconceptions on the part of some that cognition plays no role in a CLA, intentions could be viewed as the most important individual constraint (Kelso 1995). Learner’s intentions have the power to act as a specific informational constraint related to their overarching goals and could lead to stabilisation or destabilisation of existing system organisation. Intentionality is, therefore, a central constraint for practitioners to consider and frames the selective openness and responsiveness of learners to search for and select from the rich landscape of available affordances’		Renshaw and Chow [46]—‘the mutuality of the individual and environment emphasises that the individual is a perceiver of the environment and a behaver in the environment. Hence, what we see in our environment, determines what we do. What we see is dependent on what resources (i.e. parks, courts, empty spaces) are available in that environment and then upon our ability to pick-up that information. This is a key concept for practitioners as it highlights the importance of designing learning environments that provide learners with opportunities to attune to information from the environment to which they can couple their actions’
Knowledge	Cordovil et al. [48]—‘information is a physical variable available to constrain behaviour in the environment (Turvey, 1992). To detect such information, the performer needs an intrinsic metric that is specified by dimensions of his or her system. That is, the performer perceives properties of the environment, not in extrinsic units (such as metres, inches, etc.), but in relation to his or her body or body parts dimensions, his or her own action capabilities, and his or her spatial location relative to other important objects, surfaces, and people in the environment.’		Cordovil et al. [48]—‘In future research, other factors might be analysed as powerful constraints on emergent decision-making behaviour, including:... (iii) previous knowledge that the attacker and defender have of each other (i.e. understanding of relative strengths and weaknesses of the behaviour of specific opponents)’
	Roberts et al. [44]—‘the learners in the practice environment will endeavour to make sense of the chaos they are presented with by forming performance solutions via goal-directed behaviour.... well-structured environment design must offer learners the opportunity to move beyond “what” they must do, and towards an understanding that allows them to construct for themselves the “how, why, where and when” of movement.’		Roberts et al. [44]—‘An effective environment in this context will provide the learner with the opportunity to develop functional perception–action couplings or emergent synergies (coordinated states) needed to achieve the task goal.... A learner’s interactions with teammates and opponents within an environment will have the biggest impact on exploring inherent self-organisation tendencies. As learners attempt to achieve their task goal, they must collaborate with their teammates by self-organising to satisfy interacting constraints.’

Examining cognition, Chow et al. [40] stated that inference and cognitive processing were not required for direct learning within the perceptual-motor system, whilst also referring to the attention and intentions of the learner, which contradicts the lack of cognitive processing and direct nature of movement self-organisation. Likewise, Renshaw and Chow [46] explained intention and cognition as the frames through which exploration takes place, despite their mechanistic explanation referring to individuals being perceivers *of* and *in* the environment. Indeed, they even stated that ‘what we see in our environment, determines what we do’ (p. 106), which again opposes the idea that what performers bring to an environment (i.e. the cognitive frame) also impacts on what they see *and* how they perform (cf. [47]).

Finally, regarding knowledge, Cordovil et al. [48] expressed that internal qualities (intrinsic metric or representation) of performers *are* important for using environmental information. Information is described as needing to be understood relative to a performer’s own body dimensions, action capability, technique and the game context. These attributes seem to complement ideas emanating from cognitive approaches, as does the authors’ recommendation for research examining previous knowledge of opponents’ performance. Yet, their interpretation of these interactions is that they are emergent rather than—at least in part—understood, intentional and planned. Returning to the previous quotation by Roberts et al. [44], it is explained that children need to develop knowledge and understanding in PE, but that practice design needs to be underpinned by emergent and self-organisation processes such as perception–action couplings. In summary, whether reading one article or several from an ecological dynamics perspective, the narrative to date has been inconsistent, confusing and contradictory. So, one important question for practitioners and practice is: how *does* it work?!

## Second Concern: An Apparent Disregard for a Substantial Literature in Learning

As pracademics motivated to support the learning needs of individuals, it makes sense to consider the nature of learning across several domains to identify areas of alignment and/or justified difference. In this regard, although education literature has predominantly focused on traditional school subjects (e.g. maths, reading and science), learning and teaching research can offer several insights on general pedagogical/didactical principles and methods. First, rather than *one* method, varieties of methods seem to underlie optimal teaching and learning. As put by Peterson et al. [49], ‘if the “science” of pedagogy is in identifying the mechanisms and potential impacts of different approaches, the “art” is employing and combining pedagogies effectively to achieve the desired effect in context’ (p. 12). This reflects work in sport involving expert coaches and their professional judgement and decision-making (PJDM; [50]) as key to selecting and implementing appropriate approaches to suit the learner, context and situational demands. In other words, the PJDM approach (in line with Ranganathan and Driska’s [10] call for pluralism when theory is inconclusive) proposes ‘practice through theory’ where practitioners aim to understand the problem, consider the evidence, and then select an appropriate solution, rather than trying to fit the presenting issues to one single, pre-selected theory.

Second, despite advocating a variety of principles and methods, meta-analyses have provided insights into learning and teaching with evidence indicating that teachers’ ability to provide cognitively active learning situations is associated with better learning outcomes (e.g. [51, 52]). Such active learning situations are promoted by cognitively challenging tasks, activation of learners’ pre-knowledge, providing learning objectives and feedback, encouraging reflection, and questioning (e.g. [53–55]). By using these methods, educators scaffold the learning process and aid learners to develop understanding of the to-be-learnt content.

Another important insight relates to development of autonomous learners and self-regulated learning (i.e. knowing how to learn), which is believed to primarily depend on students’ motivation, cognition and metacognition [51]. Metacognition, which is considered the most important of the three (e.g. [53]) and associated with enhanced teaching effectiveness [51], can be classified into two dimensions: metacognitive *knowledge* (i.e. knowledge of one’s own knowledge and thinking) and *skills* (e.g. the ability to regulate one’s own thinking). It is not difficult to see relevance in understanding one’s current knowledge/skills—and strategies to improve these—and the ability to plan, monitor and evaluate actions for implementing self-regulated learning. Given that such learner-centred discourse is a tenet of ecological dynamics (e.g. ‘a hands-off role by allowing learners enough time and space to explore and discover appropriate movement solutions for themselves’ [45, p. 122]), it is noteworthy that limited attention has been directed toward the role of (meta)cognition within ecological dynamics. For example, even though it is claimed that ecological dynamics ‘recognises the powerful roles played by knowledge, cognitions and intentions and how these processes are deeply intertwined and integrated’ [45, p. 74], ecological dynamics has not sufficiently addressed or operationalised metacognition.

Of course, although general learning and teaching research offers these insights on pedagogical principles and methods, there may be some differences for motor learning. At the same time, it would seem rather far-reaching if these principles did not transfer in some way, as the idea that our brain has two orthogonal learning systems is unsupported by evidence. This is quite apart from the volume of literature which supports the importance of cognition and understanding in sports performance (e.g. [56–61]). So, concerning the relevance of ecological dynamics to alternative scientific literature on learning, once again, the picture, the literature and the practitioner guidance seem confused.

## Third Concern: Are the New Insights Really New?

Concern must be raised over the extent to which coaching methods are presented as novel merely because alternative explanations from the scientific literature are being proposed. As one example, many ideas presented appear to have since been contextualised and ‘packaged’ as ecological dynamics innovations [cf. 11]. Indeed, Renshaw et al. [62] stated that ecological dynamics methods represent a ‘new pedagogical framework in physical education’ (p. 119). In contrast, as experienced applied practitioners, we would suggest that manipulating environmental and task conditions is not new to coaches but represent what many have *long* regarded as ‘good practice’. Although not referred to as ecological dynamics, the use of conditioned games and drills forms a significant part of early classic coaching texts, including football in 1946 [63] or coaching rugby in 1976 [64]. Renshaw et al. [62] even highlighted anecdotal evidence of Nick Faldo’s first golf coach instructing him to hit shots with different curvature during practice, which represents a form of golf-relevant contextual interference or self-exploration of the environment. Moreover, we challenge the novelty in promoting appropriately sized implements (e.g. a junior golfer using proportionally scaled golf clubs) from ecological dynamics perspectives, as such factors were apparent within coaching texts in the 1960s [66]. Outside of the sporting context, examples are also commonplace in domains such as aviation, whereby simulation training of critical in-flight events are used to enhance judgment and decision-making skills in Air Force trainee pilots, thus demonstrating knowledge and appreciation of ‘representative design’ characteristics outside of ecological dynamics [65]. Accordingly, Harvey et al. [66] have argued that core propositions from ecological dynamics (e.g. representative games, modifying game constraints, etc.) represent ‘old wine in new bottles’ (p. 174) through the sharing and assimilation of existing and established ideas.

Another concern reflects the basis of arguments made to support the originality and benefits of the ecological dynamics approach. In this regard, ecological dynamics literature compares the advantages of this approach to what can only be described as a non-existent, or rarely occurring, ‘straw man’. In other words, what is often described as ‘traditional coaching’ is both extreme and unrepresentative of our experiences. As another example, consider the recent paper by Myszka and colleagues [69] which, some years after the writings and films of the famous martial artist Bruce Lee, actually claimed Lee’s views aligned with the ecological dynamics approach with some direct quotations even altered from his actual statements to fit their argument (cf. [25]). *If* these approaches *are* novel, then we suggest that there also needs to be consideration of and explanation for pre-existing practices, known to be effective for learning and elite performance (and indeed, still in common usage).

## Fourth Concern: Limited Empirical Evidence

As a potential consequence of the aforementioned concerns relating to clarity, universality and novelty, there is a recognised paucity of underpinning, hypothesis-driven research [67, 68] and limited empirical evidence to explain key mechanisms (e.g. attunement). To date, most articles have related to opinions or discussions about how the ever-evolving concepts could be applied or do apply to different contexts; in short, offering *alternative* explanations. Notably, however, there has been limited empirical investigation using appropriate measures to capture the key processes (e.g. cognition, task dynamics, or weather conditions) that may underpin reported outcomes. Indeed, methodologies and/or discussions have been narrow in their scope (e.g. descriptive with singular measures and limited comparison [67]) and have not always considered other pertinent variables[25, 69]. For instance, conceptual inconsistencies, such as the evolving acceptance of cognition as a contributor to performance (e.g. [70]), make comparisons with previous studies focussing on measuring movement kinematics impossible (e.g. [71]). Notably, perhaps, is the absence of neuroscientific data which has become increasingly more prominent within motor control research over the previous four decades (e.g. [72–74]). At the very least, a mixed-methods approach would go some way to verifying the veracity of explanations offered within the ecological dynamics perspective. Without substantiating or triangulating concepts or processes through empirical investigation, the potential for impact of the ecological dynamics approach is reduced due to its inability to supplant current best practice(s). Consequently, suggestions of applied practice from this approach represent only alternative explanations.

## Fifth Concern: Is Ecological Dynamics Alone Sufficiently Parsimonious? The Case of Skill Refinement

To date, the only formalised framework to offer guidance on the important process of skill refinement is the Five-A Model [75–77], which was derived *for* sport by drawing upon relevant theory and practice. To complete the *entire* process with long-term permanence and pressure resistance is a complex interdisciplinary process involving, for instance, existing automatisms governing movement control [78], alongside the systems, culture, support [77] and health issues [79] surrounding an athlete within a given sport. One key element is the use of *contrast drills* (e.g. [80–85]) as an early manipulation of task constraints. Briefly, contrast drills are a form of practice schedule where the athlete alternates between executing already existing versions of technique and the closest approximation of a desired new version. Therefore, constraints are manipulated but in a notably unrepresentative way.

Interpretation from ecological dynamics would, we presume, explain the purpose as destabilising existing movement dynamics to allow new patterns to emerge as a *direct* perceptual process [86], perhaps similar to a phase transition [87]. In other words, self-organisation enables transition from one attractor state toward another more optimal location on the perceptual-motor landscape. Mechanistically, this approach offers insight into behavioural adaptations necessary to achieve long-term permanent change and is supported by evidence that individually preferred movement patterns become increasingly stable with learning (cf. [88]).

Crucially, however, this approach does not consider cognitive mechanisms. According to Carson and Collins [75], it is important to *consciously* distinguish, or drive a ‘wedge’ between, the existing and desired technique as an initial step toward preventing future kinematic regression. Notably, research suggests that the refinement of movement skills is ineffectual without an increase in, usually at that stage, unfamiliar bodily awareness [89]; consequently, there is need for both behavioural *and* cognitive perturbation in the athlete. Indeed, athletes’ declarative understanding of ‘what is going on’ during movement (i.e. their representation) is an essential component in optimising expected behavioural effects [90]. In practice, this might involve the coach asking: ‘How did that feel?’, ‘What was different between those two executions?’ and ‘Was that better or worse?’. In short, perturbation must come from *two* sources: the new movement being realised by the athlete *and* increased conscious control over the existing kinematics. If athletes are capable of relating necessary changes to previous experiences, that may serve to enhance this process and accelerate changes, even when associated movements have been trained in the absence of representative conditions (e.g. when contrasting hockey to golf). Indeed, failure to change with the desired effect has been reported following implicit training methods, where the athlete is not consciously aware that they are making a change [91]. As such, manipulating task constraints only provides *part* of the solution.

Of course, we are not suggesting that *every* component of the skill is centrally controlled, but that control is a *blend* of top-down and bottom-up processes with automaticity existing as a gradual construct (i.e. not all-or-nothing) across the different components. Notably, recent studies exploring structural movement organisation [92] have shown motor control to differ *within* the motor system during the awareness process [93]. Moreover, research concerning optimal performance states has also challenged the traditional cognitive perspective [8] that an athlete will *always* perform under total subconscious control, depending on the motoric demands [94], task complexity [95] and/or level of consequences [96]. In short, there is increasing evidence for a mixed picture with parsimonious explanations (and more crucially, effective solutions) drawing on a combination of information-processing and ecological dynamics perspectives.

## Conclusion and Possible Next Steps

In this paper, we have critically evaluated the ecological dynamics approach and examined its clarity and coherence. To be clear, we support elements of ecological dynamics but suggest that it is insufficient as a *sole* means to guide applied practice. This is highlighted, in part, by its inability and inconsistency in addressing key concepts that underpin common practice in sport (e.g. game planning or imagery), which should represent a key consideration (see [5]). On this basis, we welcome new approaches that avoid either/or perspectives (e.g. [97, 98]) and instead draw upon pertinent empirical evidence and relevant features of various approaches (e.g. information processing or predictive processing) to address real-world issues and offer more parsimonious explanations for skill acquisition/performance (see [99], as an example). Notably, such ‘mixed pictures’ have also emerged when fundamental ecological dynamics constructs have been re-examined in performance settings (cf. [100]). Going forward, we hope to move toward a less absolutist and more practitioner-grounded approach to these important considerations.
